# Right Place, Right Function: The Contribution of Subcellular Localization to the Physiological Impact of TRPM2 Channels

**DOI:** 10.3390/life16101761

**Published:** 2026-10-21

**Authors:** Alice Hasselsweiller, Bich-Thi Camille Nguyen, Mayar Soussi, Dimitra Gkika

**Affiliations:** Univ. Lille, Inserm, CHU Lille, CNRS, U1366-UMR9020—CRCLille—Cancer Research Center of Lille, F-59000 Lille, France; alice.hasselsweiller.etu@univ-lille.fr (A.H.); bich-thi.nguyen.etu@univ-lille.fr (B.-T.C.N.); mayar.soussi.etu@univ-lille.fr (M.S.)

**Keywords:** TRPM2, subcellular localization, calcium signaling, oxidative stress, intracellular calcium homeostasis, organelle function

## Abstract

The channel TRPM2 forms cation-selective channels and is activated by redox-dynamics, including oxidative stress. Initially described at the plasma membrane, TRPM2 has been found to also localized to intracellular compartments, i.e., nucleus, mitochondria, lysosomes, and the endoplasmic reticulum. Its subcellular distribution appears to influence the Ca^2+^ signals and signaling pathways associated with each compartment, resulting in distinct cellular outcomes. In cancer, TRPM2 has been implicated in proliferation, survival, autophagy, mitochondrial homeostasis, metabolism, inflammation, and cell death. Notably, TRPM2 can exert opposing effects depending on the cellular context, oxidative state, stimulus, and subcellular localization. These findings suggest that the functional diversity of TRPM2 may arise, at least in part, from its spatial organization within the cell.

## 1. Introduction

TRPM2 (Transient Receptor Potential Melastatin 2) is a member of the TRP (Transient Receptor Potential) superfamily of ion channels, a larger and more composite class of cation channels that detect a wide range of physical and chemical stimuli, including temperature, osmolarity, and oxidative stress [1]. Among the members of the TRPM family, TRPM2 stands out due to its ability to detect oxidative stress and is defined as a significant redox-responsive ion channel that connects the production of reactive oxygen species (ROS) with intracellular Ca^2+^ signaling [2,3]. Because of this function, TRPM2 participates in the regulation of the majority of cellular processes, like cell survival, inflammation, autophagy, and mitochondrial homeostasis. As to its role of being an oxidative stress sensor, dysregulated TRPM2 activity is connected with a number of pathological conditions associated with excessive ROS production, such as neurodegenerative diseases, ischemia, vascular disorders, diabetes, infections, and cancer [3,4,5]. 

TRPM2 is a Ca^2+^-permeable, non-selective cation channel that can allow the passage of calcium ions, and the function of this channel is activated by ADP-ribose. This channel plays an important role in detecting oxidative stress, thus linking ROS and intracellular Ca^2+^ signaling [3,6]. Due to its nature as a non-selective cation channel, TRPM2 is able to conduct a lot of monovalent and divalent cations, including sodium (Na^+^), potassium (K^+^), calcium (Ca^2+^), and magnesium (Mg^2+^). According to electrophysiological examination, K^+^ is permeable through TRPM2 at a ratio of 1.1 relative to Na^+^ (PK^+^/PNa^+^), Ca^2+^ is permeable at 0.9 (PCa^2+^/PNa^+^), and Mg^2+^ is permeable at 0.5 relative to Na^+^ (PMg^2+^/PNa^+^). These parameters make TRPM2 influence both the depolarization of the cell membrane and Ca^2+^ homeostasis within cells [7,8]. 

Mechanistically, oxidative stress promotes the generation of ADP-ribose (ADPR), which binds to the MHR1/2 domain in the N-terminus, as well as to the C-terminal NUDT9-H domain of TRPM2, inducing conformational changes that favor channel opening. Additionally, Ca^2+^ binds to the intracellular regulatory binding site situated at the junction between MHR1/2 and MHR3 regions and serves as a crucial co-factor in activation of the channel (Figure 1). As a primary sensor of oxidative stress, TRPM2 is known as an important mediator of ROS-dependent signalization in many diseases that are characterized by large ROS activation, including ischemia–reperfusion injury, diabetes, inflammation, and cancer. This activation pathway plays a significant role in various clinical conditions of excessive ROS release, including ischemia–reperfusion injury, when TRPM2-induced Ca^2+^ overload leads to malfunction of cellular and tissue processes. In the central nervous system, TRPM2 activation is important for neuronal cell death after oxidative damage, including that which occurs in stroke or neurodegenerative diseases. In parallel with this, in the immune system, where TRPM2 is active in macrophages and neutrophils, it regulates the production of cytokines and chemotaxis in oxidative stress conditions. Additionally, TRPM2 is involved in dysfunction of pancreatic β-cells in diabetes when chronic oxidative stress changes the homeostasis of Ca^2+^ and insulin production. TRPM2 has interested many researchers since its activation in cancerogenesis is associated with regulation of proliferation, survival, mitochondrial function, and autophagy [9]. TRPM2 activation has been associated with neuronal cell death due to oxidative challenges in the central nervous system, like those occurring in strokes or neurodegenerative disorders [10]. At the same time, TRPM2 also plays an important role in the cascade of events associated with immune responses due to its involvement in the control of the production of cytokines and chemotaxis in immune cells like neutrophils and macrophages under oxidative stress [11]. Furthermore, TRPM2 is related to pancreatic ß-cell injury under diabetic conditions because chronic oxidative stress leads to the malfunction of Ca^2+^ homeostasis and disturbances in insulin secretion [12]. Moreover, TRPM2 is attracting much attention in the field of cancer biology, where its involvement in cellular responses due to oxidative stress has been shown to influence the proliferation of cells, their survival, mitochondrial function, autophagy, and efficacy of cellular stress-resistant mechanisms for different types of tumors. Studies on pharmacological inhibition or genetic silencing of TRPM2 showed a decrease in cell proliferative capabilities, subsequent alteration of survival pathways at the cellular level, and an increase in DNA damage accumulation [13]. 

While these pathological roles are largely driven by TRPM2 activation at the plasma membrane in response to oxidative stress, increasing evidence suggests that its functional diversity is further shaped by its subcellular localization, which enables compartment-specific Ca^2+^ signaling within intracellular organelles. Beyond its canonical localization at the plasma membrane, accumulating evidence indicates that TRPM2 is also present in intracellular compartments, where it contributes to the regulation of intracellular Ca^2+^ signaling. In particular, TRPM2 has been reported to localize to mitochondria [14], lysosomes [5], and the nucleus [15]. This compartmentalized distribution suggests that TRPM2 may exert context-dependent functions beyond its classical role as a plasma membrane ion channel, influencing broader cellular processes linked to Ca^2+^ homeostasis and cellular stress responses [16]. In particular, intracellular TRPM2 has been implicated in the regulation of organelle function and intracellular signaling pathways, highlighting its potential role in maintaining cellular homeostasis under physiological and pathological conditions.

This multiple localization allows TRPM2 to generate both global and compartmentalized Ca^2+^ signals, critically shaping cellular responses to stress. Functionally, TRPM2 has been implicated in a wide range of physiological and pathological processes, including inflammation, cell death, neurodegeneration, and cancer [16]. Notably, its role appears to be highly context-dependent, as TRPM2 can exert both protective and deleterious effects depending on the cellular environment and intensity of stress stimuli, highlighting its emerging importance as a multifaceted regulator and potential therapeutic target [17]. 

For example, continuous activation of TRPM2 may lead to apoptosis or necrosis that occurs under exposure to strong oxidative stress. Meanwhile, moderate TRPM2 activation has been shown to promote cell survival due to adaptive response required for normal redox balance in the cells.

These diverse, context-dependent roles raise a fundamental question: how can a single channel produce such divergent outcomes? We first outline the structural and functional properties of TRPM2, then dissect the molecular mechanisms through which it senses oxidative stress. We then propose that the answer lies largely in space: the compartment-specific distribution of TRPM2 determines which downstream pathways its activation engages, transforming a common Ca^2+^ signal into distinct cellular responses.

## 2. Structure Features and Activation Mechanisms of TRPM2

### 2.1. Structure and Domains

TRPM2 was identified in 1998 through the cloning of a novel member of the transient receptor potential channel family, initially described as LTRPC7 (Long Transient Receptor Potential Channel) [18]. TRPM2 is also known as LTRPC2 (Long Transient Receptor Potential Channel), KN3, and EREG1 (Estrogen-Responsive Element-Associated Gene 1) [17,19]. In humans, the TRPM2 gene is located on chromosome 21 (21q22.3), and the corresponding protein has approximately 1’503 amino acids. The TRPM2 gene spans approximately 90 kb and consists of around 32 exons. The TRPM2 channel belongs to the TRPM subfamily and displays the common structure of TRP channels, consisting of six transmembrane segments with a pore-forming loop between the fifth and sixth segments. 

The N-terminal region of TRPM2 consists of a significant cytoplasmic domain that is essential for the configuration and operation of the channel. Like other members of the TRPM family, this region includes a collection of conserved structural motifs, which are important for the architecture of the channel. In particular, the N-terminal region features four melastatin homology regions (MHR1-4), which are specific characteristic features of TRPM2 channels and are involved in interactions between subunits and regulation of channel operation. Structural analyses have demonstrated that the N-terminal melastatin homology regions contribute significantly to the intracellular setup of TRPM2, as they participate in the assembly of the tetramer channel. Moreover, a conserved Zn^2+^-binding site has also been identified in the N-terminal region, which is believed to be crucial for maintaining the structure and functioning of the ion channel [20]. MHR domains are thought to contribute to the stabilization of the channel structure and to the transmission of conformational changes from ligand-binding sites to the pore region [14]. In this context, the N-terminus acts as an important regulatory hub that integrates intracellular signals and modulates channel activity.

Besides the conserved melastatin homology regions, the N-terminal domain of TRPM2 harbors an IQ-like motif, a short sequence that typically mediates binding to calmodulin and other Ca^2+^-binding proteins. This motif maps to the MHR3 domain. A second IQ-like motif lies within the C-terminal NUDT9-H domain, which suggests that calmodulin-dependent regulation may engage more than one region of the channel. Although this second motif departs from the canonical IQ consensus, it may still contribute to Ca^2+^-dependent regulation of TRPM2. Since intracellular Ca^2+^ is needed for full channel activation, an IQ-like motif in the N-terminus could allow TRPM2 to sense changes in cytosolic Ca^2+^ directly [21]. Such a motif could couple Ca^2+^ binding to conformational changes in the channel and thereby tune its gating.

The C-terminal region of TRPM2 contains a conserved TRP box motif, immediately downstream of the sixth transmembrane segment. This short sequence, a hallmark of TRP channels, contributes to channel gating and to the structural coupling of the transmembrane domain with the cytosolic regions. The TRP box is thought to relay the conformational changes required for channel opening, thus coupling ligand-induced activation to pore gating [5].

Besides the TRP box, the C-terminal domain of TRPM2 includes a coiled-coil region that contributes to subunit assembly and channel stabilization. This structural motif mediates protein–protein interactions between individual TRPM2 subunits, thereby supporting the formation and maintenance of the functional tetrameric complex. Consistent with this role, deletion of the coiled-coil domain has been shown to severely disrupt subunit interactions and markedly reduce ADPR-evoked channel currents [22] (Figure 1).

Beyond these structural elements, the C-terminal region of TRPM2 also harbors a Nudix homology domain, termed NUDT9-H. This domain shares sequence similarity with the mitochondrial ADP-ribose pyrophosphatase (ADPRase) NUDT9 and plays a central role in channel activation [23].

### 2.2. Activation and Ligands

TRPM2 activation is best understood as a cooperative allosteric gating process in which cytosolic ligand binding, intracellular Ca^2+^ sensing, and pore-domain rearrangements jointly control channel opening. Structural studies indicate that TRPM2 is organized into distinct functional layers: a ligand-sensing layer formed by the MHR1/2 and NUDT9-H domains, a linker layer composed of the MHR3/4 domains, and the transmembrane pore domain responsible for ion conduction. In the basal state, in the absence of ADPR and Ca^2+^, the channel remains closed through stabilizing interactions between the N-terminal MHR domains, the C-terminal NUDT9-H domain, and the transmembrane pore [16] (Figure 2A). Activation is initiated when intracellular ADPR binds to the high-affinity MHR1/2 domain (Figure 2B), which induces conformational rearrangements that shift the channel into a primed intermediate state [24]. These changes are relayed through the MHR3/4 linker layer toward the transmembrane region, repositioning the TRP helix and relieving the constraint imposed by the S4–S5 linker, which ultimately favors opening of the S6 activation gate. However, this primed configuration does not fully open the pore; it instead repositions cytosolic domains so that they can couple effectively to downstream gating elements. Full channel activation requires intracellular Ca^2+^ (Figure 2C), which acts as an essential co-agonist by stabilizing the open conductive conformation and strengthening coupling between cytosolic domains and the pore. Consistent with this, Ca^2+^ was shown to bind at a conserved regulatory site formed by residues Glu843 and Gln846 within TMS2, Asn869 in TMS3, and Glu1073 on the TRP helix [25]. 

Additional modulators, including cADPR, ROS-dependent metabolic pathways, and temperature, further tune this process by increasing ligand sensitivity and promoting conformational transitions. cADPR is a weak TRPM2 agonist with low potency and efficacy: it requires high, non-physiological concentrations to evoke minimal channel activity compared with ADPR [26]. It nevertheless acts as a strong positive modulator, markedly increasing ADPR sensitivity and shifting TRPM2 activation to much lower ligand concentrations. This points to a synergistic effect on channel gating, although its binding site and mechanism remain unclear. By contrast, ROS, including H_2_O_2_, and oxidants such as cumene hydroperoxide (Cpx) activate TRPM2 predominantly through an indirect route, by stimulating PARP-dependent ADPR production, although minor ADPR-independent effects have been reported. H_2_O_2_ induces diffuse intracellular oxidative stress and DNA damage, whereas Cpx, which is more lipophilic, primarily causes membrane lipid peroxidation. Despite these differences, both oxidants ultimately converge on the same ADPR-mediated mechanism of TRPM2 activation.

Temperature also modulates channel gating by lowering the energetic barrier for conformational transitions and increasing ligand sensitivity, which points to a thermosensitive component of TRPM2 activation. Taken together, these stimuli converge on a unified gating mechanism in which ADPR-dependent rearrangements of the cytosolic domains, potentiated by Ca^2+^ binding and modulated by ROS, cADPR, and heat, cooperatively drive pore opening. This positions TRPM2 as an integrated sensor of metabolic stress and cellular homeostasis.

After activation, TRPM2 displays a stable, linear current–voltage (I–V) relationship with a reversal potential near 0 mV, a signature of a non-selective cation channel permeable to both monovalent and divalent ions, including Na^+^, K^+^, Ca^2+^, and Mg^2+^ [27]. Single-channel recordings likewise show nearly linear conductance, ranging from approximately 52–60 pS at negative potentials to ~72 pS at positive potentials [27]. Functional analysis has identified several residues within the selectivity filter that critically control ion permeation, including Gly960, Gly981, Ala987, and Gly1022, which contribute differently to Ca^2+^ and Mg^2+^ permeability [7]. In addition, whereas whole-cell TRPM2 currents remain stable in the continuous presence of ADPR and Ca^2+^, excised patch recordings show a rapid, time-dependent decline in current amplitude, termed rundown, which reflects a progressive destabilization of the open conductive state [16]. Mechanistic studies have shown that this rundown arises from conformational rearrangements within the selectivity filter that gradually reduce the number of active channels [28]. Notably, introducing negatively charged residues within the pore region markedly stabilizes channel activity and prevents complete inactivation, yielding non-inactivating TRPM2 variants that retain normal ADPR/Ca^2+^-dependent gating but display sustained maximal currents. These findings highlight the central role of the selectivity filter in both ion permeation and the structural stability of the conductive state, making the pore region a critical determinant of TRPM2 gating dynamics and pharmacological modulation.

Accumulating evidence indicates that TRPM2 can exert distinct cellular functions depending on its subcellular localization. While plasma membrane TRPM2 primarily mediates extracellular Ca^2+^ influx in response to oxidative stress and the production of ADPR, intracellular pools of TRPM2 have been linked to compartment-specific signaling pathways. In this review, we describe the different subcellular localizations of TRPM2 and examine how these compartment-specific distributions shape the diverse physiological and pathological functions of this channel.

## 3. How Does Subcellular Localization Shape TRPM2 Function?

### 3.1. Plasma Membrane

Although this review focuses mainly on intracellular organelles, TRPM2 functions at the plasma membrane will be briefly described here through four examples: ischemia, diabetes, inflammation, and cancer. TRPM2 is widely expressed at the plasma membrane of various cell types, such as cardiomyocytes, neurons, and immune cells (monocytes, macrophages, and neutrophils, as mentioned above). In these cells, TRPM2 serves as a Ca^2+^ influx pathway [29]. According to Perraud et al. 2001 [27], TRPM2 was shown using patch-clamp in the whole-cell configuration, showing the influx of Ca^2+^ in HEK-293 cells, and this was later confirmed via immunostaining of TRPM2 in monocytes [30]. Altogether, these studies unveil TRPM2 as a channel present on the plasma membrane.

Regarding ischemia, and more specifically ischemia–reperfusion (I/R) injury, which **is** characterized by massive ROS production and intracellular Ca^2+^ overload, activation of plasma membrane TRPM2 contributes to tissue damage and cell death [9]. Indeed, TRPM2 links ROS production to the Ca^2+^ overload observed in I/R injury [9,31]. Upon activation, TRPM2-mediated Ca^2+^ influx amplifies oxidative stress, impairs mitochondrial function, and triggers apoptotic signaling, including caspase activation, in a tissue-dependent manner. Given its involvement in I/R injury in major organs such as the brain, heart, kidney, and liver, TRPM2 represents a promising therapeutic target [3].

In diabetes, the hyperglycemic environment promotes oxidative stress in cardiomyocytes, and the resulting increase in ROS production is associated with higher TRPM2 expression [32]. Hu et al. 2024 [32], showed that TRPM2 upregulation was linked to the high-glucose environment in a mouse model of diabetic cardiomyopathy (DCM). In this context, the MEK/ERK pathway was also activated, Bax and cleaved caspase-3 levels increased, and levels of the anti-apoptotic protein BCL-2 decreased. DCM was also characterized by impaired autophagy, as shown by increased p62 levels, a reduced LC3-II/LC3-I ratio, and downregulation of the mTORC1 pathway. TRPM2 silencing attenuated, and even partially reversed, these hyperglycemia-induced alterations: it reduced cardiomyocyte apoptosis, restored autophagy markers, and improved cardiac function in diabetic mice [32], thus making TRPM2 an important contributor through the MEK/ERK and mTORC1 pathways.

In addition, TRPM2 opening at the plasma membrane promotes NLRP3 inflammasome activation in macrophages [33]. Oxidative stress induces mitochondrial ROS production, which in turn activates TRPM2 and triggers Ca^2+^ influx. The resulting rise in intracellular Ca^2+^ drives NLRP3 inflammasome activation, leading to caspase-1 activation and the secretion of mature IL-1β, and ultimately to pyroptosis [34].

Under intensive oxidative stress, the outcome shifts from inflammation to cell death. Excessive TRPM2 activation causes intracellular Ca^2+^ overload, which disrupts Ca^2+^ homeostasis and triggers apoptotic cell death in several cell types, including hepatocytes, neurons, and endothelial cells [2].

By contrast, this response appears to be altered in several cancers, including breast, prostate, pancreatic, gastric, and lung cancer. In these cells, TRPM2 is frequently overexpressed at the plasma membrane, sustaining Ca^2+^ influx and thereby supporting pro-survival functions. This enhanced TRPM2 activity promotes tumor cell proliferation and contributes to chemoresistance [4,17]. Overall, the consequences of TRPM2 activation appear to be highly context-dependent, shaping cell fate and making TRPM2 a promising therapeutic target in oncology.

Moreover, TRPM2 localization can be regulated depending on the cell type and its cellular environment. Kheradpezhouh et al. 2018 [35], demonstrated via Western blotting and immunofluorescence staining that TRPM2 is predominantly localized within intracellular compartments in hepatocytes. In control hepatocytes, TRPM2 was barely detected at the plasma membrane, with only 3.57 ± 0.47% of the channel found at the cell surface. Strikingly, after H_2_O_2_ treatment, this proportion increased to 16.17 ± 0.73%, a more than fourfold rise. These findings indicate that TRPM2 trafficking is dynamically regulated in response to oxidative stress and suggest that oxidative stress promotes TRPM2 translocation to the plasma membrane, thereby facilitating its activation [35].

Taken together, these examples show that TRPM2 is found at the plasma membrane and that its activation triggers distinct downstream mechanisms, as illustrated by I/R injury, diabetes, and inflammation. These examples are only illustrative, however, and the functional impact of TRPM2 activation extends well beyond them. Overall, the consequences of TRPM2 opening depend on the intensity of oxidative stress: low-to-moderate exposure can activate pro-inflammatory gene programs through several signaling pathways, whereas intense oxidative stress can cause cell death by disrupting Ca^2+^ homeostasis, mainly through Ca^2+^ overload. These outcomes also vary with cellular context, as TRPM2 activation differs between healthy and cancerous cells, which adds another layer of complexity to its signaling at the plasma membrane. In addition, the oxidative stress-induced trafficking of TRPM2 to the plasma membrane provides a further level of regulation [35]. Beyond the plasma membrane, TRPM2 is also found in intracellular organelles, including the nucleus, mitochondria, lysosomes, and ER.

### 3.2. Nucleus

TRPM2 has been reported to localize to the nucleus, suggesting potential roles beyond classical ion channel function [36]. Notably, nuclear localization of TRPM2 has been predominantly described in cancer cells, where accumulating evidence suggests that it contributes to tumor progression and resistance to cellular stress. 

In breast adenocarcinoma cells, TRPM2 was detected in the nucleus via subcellular fractionation followed by Western blot analysis. Functional inhibition or silencing of TRPM2 significantly reduced cell proliferation while increasing DNA damage, as assessed using the comet assay, which points to a protective role of TRPM2 in maintaining genomic integrity in cancer cells [13]. Similarly, in oral squamous cell carcinoma (OSCC), TRPM2 nuclear localization was first identified via subcellular fractionation and Western blot analysis and then confirmed via immunofluorescence and immunohistochemical staining, which showed marked overexpression of TRPM2 in the nuclei of tumor cells compared with adjacent normal tissues. TRPM2 inhibition or silencing significantly reduced cell proliferation, migration, and invasion while increasing apoptosis and oxidative DNA damage, consistent with a pro-survival and pro-tumorigenic role [37]. The same pattern was observed in prostate cancer cells, where subcellular fractionation followed by Western blot analysis and immunofluorescence microscopy showed that TRPM2 is predominantly localized to the nuclei of malignant cells, whereas it is mainly detected at the plasma membrane and in the cytoplasm of non-cancerous prostate cells. Functional inhibition of TRPM2 selectively reduced the proliferation of prostate cancer cells with minimal effects on normal prostate cells, which further supports a cancer-specific role of nuclear TRPM2 in promoting tumor cell survival [38].

Rah et al. 2023 [39], showed that TRPM2 plays a central role in nuclear Ca^2+^ signaling by acting downstream of the CD38/ADPR axis in hepatocytes, where it mediates sustained Ca^2+^ transients required for the transcriptional activation of gluconeogenic genes such as G6PC and PCK1. Notably, this TRPM2-dependent nuclear Ca^2+^ signaling was physiologically relevant during fasting and enhanced in diabetic mice. Mechanistically, ADPR activates TRPM2 to drive nuclear Ca^2+^ influx from perinuclear Ca^2+^ stores, illustrating a compartmentalized mode of Ca^2+^ signaling that links gene regulation to metabolic disease [39]. The subcellular localization of TRPM2 in this context was demonstrated using subcellular fractionation followed by Western blot analysis and further supported by immunofluorescence microscopy with Lamin B1 as a nuclear envelope marker, indicating an enrichment of TRPM2 at the inner nuclear membrane.

Taken together, these findings indicate that TRPM2 nuclear localization is associated with important regulatory functions. Accumulating evidence suggests that nuclear TRPM2 contributes to the control of intracellular Ca^2+^ signaling, oxidative stress responses, DNA damage regulation, and transcriptional programs involved in cell survival. Although the precise mechanisms governing its nuclear targeting and function remain poorly understood, these observations highlight the emerging importance of TRPM2 as a multifunctional regulator in both physiological and pathological contexts, particularly in cancer and metabolic diseases.

### 3.3. Mitochondria

In addition to its roles at the plasma membrane and nucleus, TRPM2 has been reported to localize to mitochondria, where it may contribute to regulation of mitochondrial homeostasis, cellular metabolism, and redox balance through effects on mitochondrial Ca^2+^ signaling, dynamics and fragmentation, bioenergetic activity, ROS production, and cell death. 

In a model of cisplatin-induced acute kidney injury, B. Yu et al. 2023 [40], demonstrated that TRPM2 protects renal tubular epithelial cells by preserving mitochondrial function and limiting oxidative stress. Cisplatin-induced nephrotoxicity remains a major clinical limitation and is characterized by oxidative stress, mitochondrial dysfunction, and acute kidney injury. TRPM2 deficiency exacerbated mitochondrial dysfunction, as evidenced by increased mitochondrial ROS production, loss of mitochondrial membrane potential, and reduced ATP levels. Mechanistically, TRPM2 regulated autophagy through the AKT/mTOR signaling pathway, thereby promoting the clearance of damaged mitochondria and sustaining cellular bioenergetics under cisplatin-induced stress [40]. In this study, mitochondrial localization of TRPM2 was shown by immunofluorescence in mouse renal tubular epithelial cells, using the mitochondrial marker TOM20 as a reference, which supports its association with mitochondria in this in vivo model.

In acute myeloid leukemia (AML), S. Chen et al. 2020 [41], demonstrated that TRPM2 is essential for leukemic cell proliferation and survival. TRPM2 knockdown markedly reduced cell viability and proliferation while increasing apoptosis. Its role in mitochondrial homeostasis was supported by the finding that TRPM2 depletion increased mitochondrial ROS production, as measured with MitoSOX, while reducing oxygen consumption rate (OCR) and intracellular ATP levels. These changes were accompanied by a clear mitochondrial dysfunction phenotype, including loss of mitochondrial membrane potential (Δψm) and impaired mitochondrial Ca^2+^ uptake. In addition, TRPM2 knockdown impaired autophagic flux, resulting in defective clearance of damaged mitochondria. At the molecular level, expression of autophagy-related proteins was reduced, whereas reconstitution of ATF4 or CREB in TRPM2-deficient cells restored cell proliferation, viability, and autophagy protein expression, which indicates that TRPM2 regulates a transcriptional program critical for mitochondrial homeostasis and leukemic cell survival [41].

In triple-negative breast cancer (TNBC), we previously showed that TRPM2 is predominantly localized intracellularly, with approximately 80% of the channel detected in the intracellular compartment in both untreated and chemotherapy-treated MDA-MB-231 cells, a TNBC cell line. Using confocal microscopy in cells expressing the mitochondrial marker DsRed2-Mito, we further observed colocalization of TRPM2 with mitochondria following treatment with epirubicin, cyclophosphamide, and paclitaxel (ECP), which supports a mitochondrial localization of TRPM2 under chemotherapy-induced stress. TRPM2 knockdown reduced the expression of several mitochondrial Ca^2+^ transport components, including MCU1, MCU2, MCU3, VDAC, MCUR1, and MICU, suggesting that TRPM2 contributes to the regulation of mitochondrial Ca^2+^ homeostasis. Consistent with this, TRPM2 inhibition with tatM2NX increased both the magnitude and duration of ionomycin-induced mitochondrial Ca^2+^ responses, which suggests that mitochondrial TRPM2 promotes Ca^2+^ efflux and thereby limits mitochondrial Ca^2+^ accumulation. We therefore propose that TRPM2 may function as a mitochondrial Ca^2+^ leak channel, potentially activated by mitochondrial ROS, thereby preventing Ca^2+^ overload and supporting mitochondrial adaptation and cell survival following chemotherapy. Together, these findings identify mitochondrial TRPM2 as a potential regulator of Ca^2+^ homeostasis and chemotherapeutic adaptation in TNBC cells [42]. 

Although a direct mitochondrial localization of TRPM2 has not been demonstrated in this context, TRPM2 may nevertheless influence mitochondrial dynamics and homeostasis. In human corneal epithelial (HCE) cells subjected to scratch injury, Huo et al. 2020 [43], investigated the role of TRPM2 in EGF-induced ROS signaling and mitochondrial autophagy. Using wound-healing, EdU, and CCK-8 assays, flow cytometry, fluorescence-based analyses of mitophagy and apoptosis, and Western blotting of mitophagy- and mitochondrial apoptosis-related proteins, they showed that EGF-induced TRPM2 activation promoted mitophagy. Pharmacological inhibition of TRPM2 with clotrimazole (CLT) attenuated the beneficial effects of EGF, reducing cell migration, proliferation, and viability while increasing apoptosis. TRPM2 inhibition was also associated with altered mitophagy and changes in BNIP3, Parkin, and cytochrome c expression, which supports a role for TRPM2 in mitochondrial quality control. These findings suggest that, under oxidative stress, TRPM2 can indirectly influence mitochondrial homeostasis by promoting the removal of damaged mitochondria through mitophagy, thereby contributing to cellular repair and survival [43].

Beyond its role in mitochondrial quality control, TRPM2 has also been implicated in the regulation of mitochondrial morphology and dynamics under metabolic stress. In endothelial cells exposed to high glucose, Abuarab et al. 2017 [44], showed, using TRPM2 pharmacological inhibition (2-APB), siRNA silencing, and TRPM2 knockout, together with live-cell imaging of mitochondrial morphology and intracellular Ca^2+^ and Zn^2+^, that TRPM2 activation promotes lysosomal membrane permeabilization and Zn^2+^ release, which subsequently results in mitochondrial Zn^2+^ accumulation and Drp1-dependent mitochondrial fission [44]. Similarly, in pancreatic β cells exposed to palmitate, Li et al. 2017 [45], reported that TRPM2 activation contributes to mitochondrial Zn^2+^ accumulation, Drp1 recruitment, and excessive mitochondrial fission, ultimately promoting β-cell death. 

In hippocampal neurons, Li et al. 2017 [46], further showed that ROS-induced TRPM2 activation causes lysosomal dysfunction and Zn^2+^ release, followed by mitochondrial Zn^2+^ accumulation, mitochondrial dysfunction, and ROS generation, thereby contributing to neuronal death [46]. Consistent with this, in SH-SY5Y neuroblastoma cells, TRPM2 inhibition prevented H_2_O_2_-induced lysosomal dysfunction, mitochondrial Zn^2+^ accumulation, fragmentation, and ROS production, which supports a positive feedback mechanism in which TRPM2 links lysosomal Zn^2+^ release to mitochondrial dysfunction and oxidative stress [47].

### 3.4. Lysosome

Lysosomes act as the terminal degradative hubs of autophagy, clearing ROS-damaged macromolecules and organelles and thereby contributing to cellular homeostasis. Yet lysosomes are not only regulators of oxidative stress but also highly susceptible targets of oxidative damage. Excessive ROS production can impair lysosomal function and trigger lysosomal membrane permeabilization (LMP), which releases cathepsins and other lysosomal contents into the cytosol and can engage several cell death pathways, including apoptosis, ferroptosis, and autosis, depending on the cellular context. This oxidative–lysosomal cross-talk has been particularly implicated in neuronal death driven by lysosomal dysfunction in neurodegenerative diseases, as well as in cancer and cardiovascular pathology. Given the vulnerability of lysosomes to oxidative damage, the presence of TRPM2 in the lysosomal membrane is particularly relevant, as it positions the channel as a potential molecular link between oxidative stress and lysosomal function. As lysosomal Ca^2+^ signaling is closely linked to the regulation of autophagy, lysosomal TRPM2 may influence autophagy by modulating local Ca^2+^ dynamics and lysosomal function. In the endolysosomal compartment, TRPM2 has been proposed to function as an intracellular Ca^2+^ release channel involved in the regulation of vesicular trafficking, autophagy, and lysosome-dependent stress responses. By shaping localized Ca^2+^ signaling, lysosomal TRPM2 may help coordinate intracellular degradation pathways and cellular adaptation to oxidative stress. 

In dendritic cells, Sumoza-Toledo et al. 2011 [48], demonstrated that both human and mouse TRPM2 localize to lysosomes, where the channel functions as an intracellular Ca^2+^ release channel activated by ADP-ribose. Using patch-clamp recordings and Ca^2+^ imaging, they found that TRPM2-mediated lysosomal Ca^2+^ release contributes to dendritic cell maturation and chemotaxis. Specifically, TRPM2 deficiency in dendritic cells markedly impaired intracellular Ca^2+^ signaling following CXCL12 stimulation, which reduced the expression of maturation markers, including CD80, CD86, and MHC-II, and impaired migration toward chemokine gradients. These findings indicate that lysosomal TRPM2 regulates dendritic cell maturation and trafficking through ADPR-dependent lysosomal Ca^2+^ signaling [48].

Lysosomes are recognized as dynamic intracellular Ca^2+^ stores that contribute to local and global Ca^2+^ signaling. By releasing Ca^2+^ into the cytosol, lysosomal ion channels can influence multiple cellular processes, including vesicular trafficking, autophagy, and cell death. TRPM2 has also been shown to function as a lysosomal Ca^2+^ release channel in pancreatic β cells. Using electrophysiological recordings and Ca^2+^ imaging approaches, Lange et al. 2009 [49], demonstrated that intracellular ADP-ribose triggers TRPM2-dependent Ca^2+^ release from bafilomycin A-sensitive lysosomal stores, an effect confirmed by pharmacological depletion of lysosomal Ca^2+^. Immunofluorescence analyses also showed partial colocalization of TRPM2 with lysosomal markers, consistent with a vesicular localization. Functionally, lysosomal TRPM2-mediated Ca^2+^ release contributed to the global intracellular Ca^2+^ response in β cells, acting together with plasma membrane TRPM2 channels to amplify Ca^2+^ signaling. Notably, both plasma membrane and lysosomal TRPM2 pools were implicated in hydrogen peroxide-induced β-cell death, pointing to a dual role of TRPM2 in cellular stress responses [49].

In vascular smooth muscle cells (VSMCs), Zhao et al. 2020 [50], showed that TRPM2 localizes to lysosomes and promotes autophagic degradation under oxidative stress conditions. Immunofluorescence analyses revealed colocalization of TRPM2 with the lysosomal marker LAMP1, which supports its lysosomal distribution. Functionally, TRPM2 deficiency impaired autophagic flux, resulting in the accumulation of LC3-II and p62-positive autophagic vesicles, which indicates a defect in autophagosome degradation rather than in autophagy initiation. The authors further reported that TRPM2-mediated lysosomal Ca^2+^ signaling is required for proper lysosomal acidification and degradative function, thereby supporting intracellular protein clearance and cellular homeostasis in VSMCs [50]. 

Further, lysosomes are not isolated organelles but form functional contacts with other intracellular compartments, particularly the ER, which allow the exchange of ions and the coordination of intracellular signaling. In this context, Kang et al. 2024 [51], highlighted the functional interplay between lysosomes and the ER in TRPM2-mediated Ca^2+^ signaling. Using Ca^2+^ imaging, pharmacological modulation of lysosomal and ER Ca^2+^ stores, and TRPM2-deficient mouse macrophages, they showed that mitochondrial ROS induced by LPS and palmitic acid activate lysosomal TRPM2, which promotes Ca^2+^ release from lysosomal stores. Notably, lysosomal Ca^2+^ depletion triggered ER-to-lysosome Ca^2+^ transfer, replenishing lysosomal Ca^2+^ stores and sustaining TRPM2-dependent signaling. This inter-organelle Ca^2+^ exchange in turn led to ER Ca^2+^ depletion, store-operated Ca^2+^ entry (SOCE), and downstream inflammasome activation. Together, these findings define an ER-lysosome Ca^2+^ signaling axis in which lysosomal TRPM2 helps coordinate inter-organelle Ca^2+^ dynamics during metabolic inflammation [51].

### 3.5. Endoplasmic Reticulum

The ER represents the major intracellular Ca^2+^ store and plays a central role in the regulation of intracellular Ca^2+^ homeostasis and signaling. Given the importance of ER Ca^2+^ dynamics in numerous cellular processes, localization of TRPM2 to this compartment would provide an additional mechanism by which the channel could shape intracellular Ca^2+^ signaling. Evidence for an ER localization of TRPM2 remains limited and partly contradictory. As mentioned in the previous paragraph, Lange et al. 2009 [49], investigated the subcellular distribution of TRPM2 in pancreatic β cells and reported little to no colocalization with ER markers, concluding that intracellular TRPM2 was predominantly associated with lysosomal compartments rather than the ER. These findings suggest that TRPM2-dependent intracellular Ca^2+^ release in β cells mainly originates from lysosomal stores rather than classical ER Ca^2+^ reservoirs. In contrast, Chen et al. 2014 [52], used subcellular fractionation and three-dimensional confocal microscopy in SH-SY5Y neuroblastoma cells and showed that both TRPM2-L and TRPM2-S isoforms preferentially associate with ER structures. Quantitative colocalization analyses revealed a stronger association of TRPM2 with the ER than with mitochondria, which suggests that ER localization may be particularly relevant in specific cellular contexts such as neuroblastoma [52]. Recent evidence further indicates that TRPM2 isoforms can differ in their intracellular trafficking and functional properties. In mouse cells, TRPM2 isoform 1, corresponding to the full-length channel, predominantly reaches the plasma membrane and mediates oxidative stress-induced Ca^2+^ influx, whereas isoform 7 is targeted to the endoplasmic reticulum-associated degradation (ERAD) pathway, which leads to its degradation and reduces the expression and activity of full-length TRPM2. These findings suggest that alternative TRPM2 isoforms may not only exhibit distinct functional properties but may also differ in their intracellular trafficking and subcellular distribution [53]. Together, these studies indicate that TRPM2 localization is highly dependent on both cellular context and isoform identity, with the ER localization and intracellular trafficking potentially contributing to the functional diversity of TRPM2.

## 4. Conclusions

In summary, accumulating evidence indicates that TRPM2 is not restricted to the plasma membrane but is also associated with several intracellular compartments, including the nucleus, mitochondria, lysosomes and the ER. As summarized in the Table 1 and Figure 3, these distinct localizations have been identified in different cellular and physiological contexts and are associated with compartment-specific effects and signaling pathways. Through these localizations, TRPM2 can influence diverse cellular processes, including autophagy, mitochondrial homeostasis, metabolic adaptation, inflammation, proliferation, and cell death. However, these intracellular localizations should not be considered universal features of cancer cells, as the available evidence derives from different tumor types, cellular models, and experimental conditions. The presence and functional significance of TRPM2 in specific compartments may therefore depend on the cellular context, its physiological or pathological state, and the stimuli investigated. It is also important to distinguish direct organelle localization from functional associations with intracellular compartments. Further comparative studies are therefore needed to establish whether these localizations are conserved across tumor types or restricted to specific contexts. Such work will be essential to clarify the context-dependent roles of TRPM2 in cancer and to assess its potential as a therapeutic target.

## Figures and Tables

**Figure 1:**
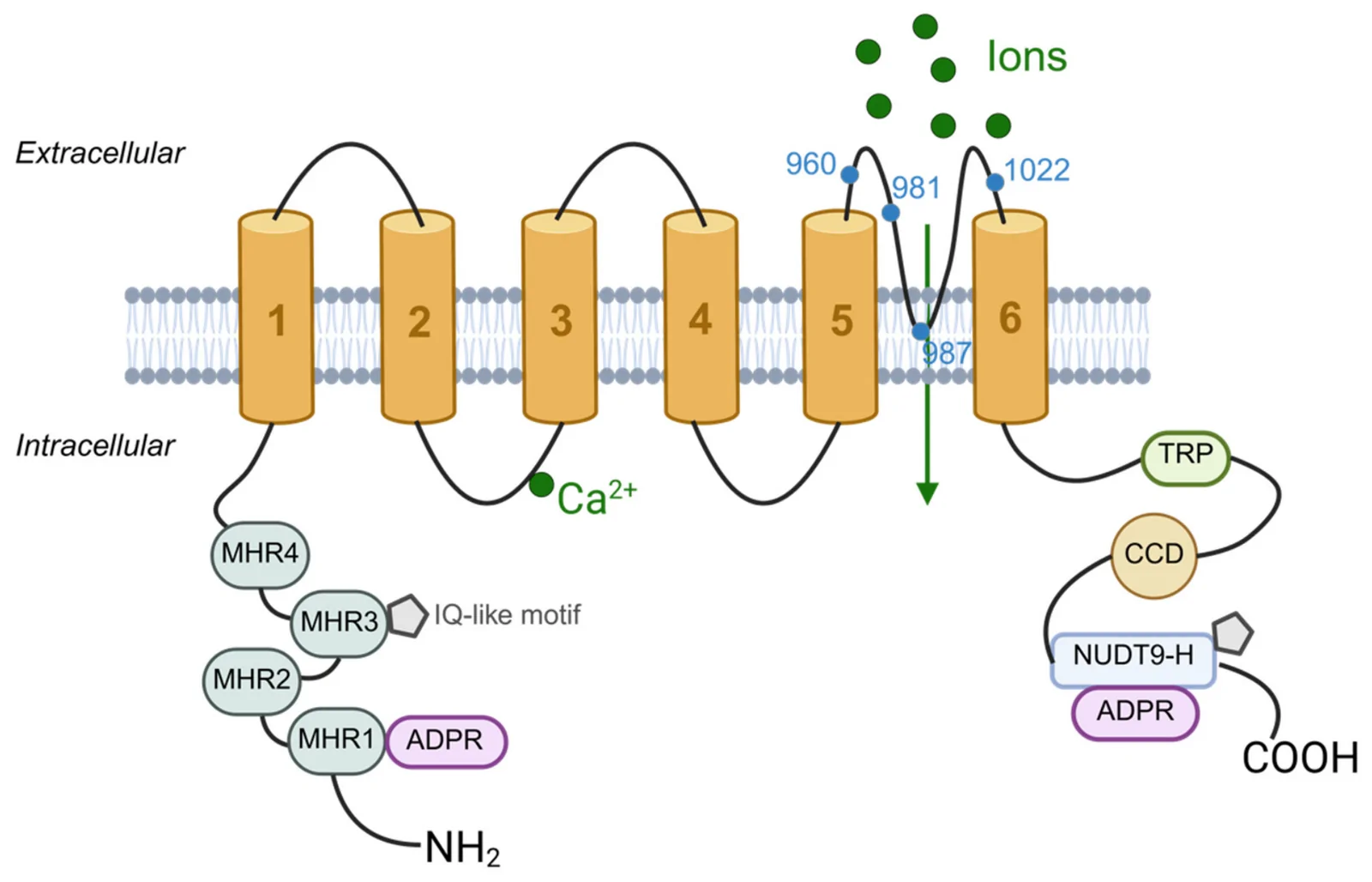
Structure of the TRPM2 channel. ADPR can bind to the MHR1/2 and NUDT9-H domains of TRPM2. Cations, including Ca^2+^, permeate through the channel pore formed by transmembrane segments S5 and S6. Gly960, Gly981, Ala987, and Gly1022 contribute to Ca^2+^ and Mg^2+^ permeability. In addition, Ca^2+^ can bind to an intracellular regulatory site located within the MHR1/2–MHR3 region, contributing to channel activation. CaM: calmodulin binding domain, TRP: Transient Receptor Potential, CCD: Coiled-Coil Domain, ADPR: Adenosine Diphosphate Ribose, NUDT9-H: Nudix ADPR pyrophosphatase Type motif 9, MHR: TRPM homology region.

**Figure 2:**
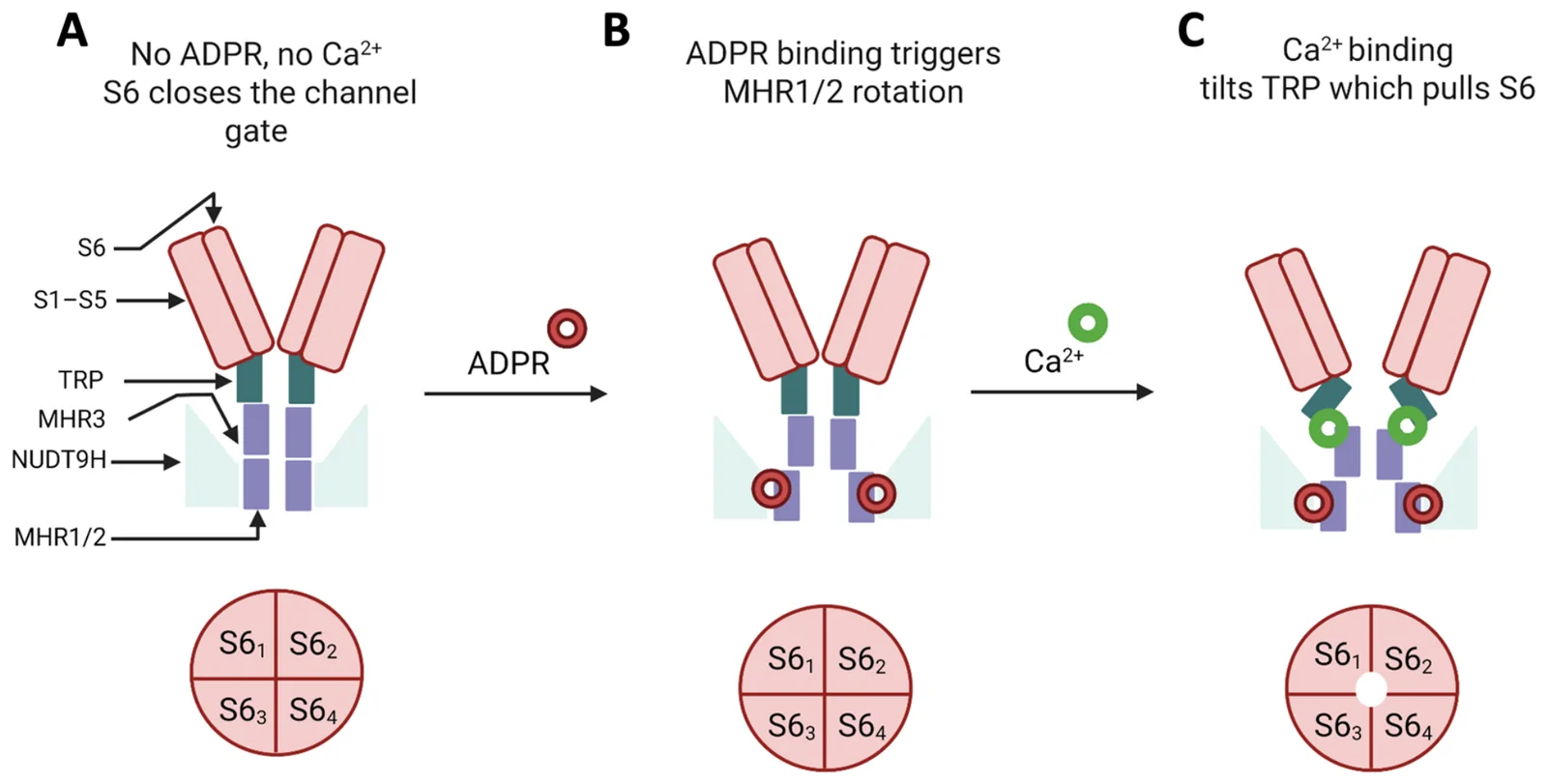
Schematic representation of TRPM2 channel gating mechanism—Adapted from [16]. (**A**) In the absence of ADPR and intracellular Ca^2+^, the channel adopts a closed conformation in which transmembrane segment S6 forms a tight intracellular gate. (**B**) Following ADPR binding, conformational rearrangements occur within the cytosolic ligand-sensing domains, including movements of the NUDT9-H and MHR1/2 regions, which promote an intermediate state characterized by partial reorganization while the pore remains closed. (**C**) Subsequent intracellular Ca^2+^ binding to regulatory sites induces further allosteric coupling, leading to mechanical displacement of S6, and rotation of the pore-lining helices, ultimately resulting in full opening of the TRPM2 homotetramer and ion conduction.

**Figure 3:**
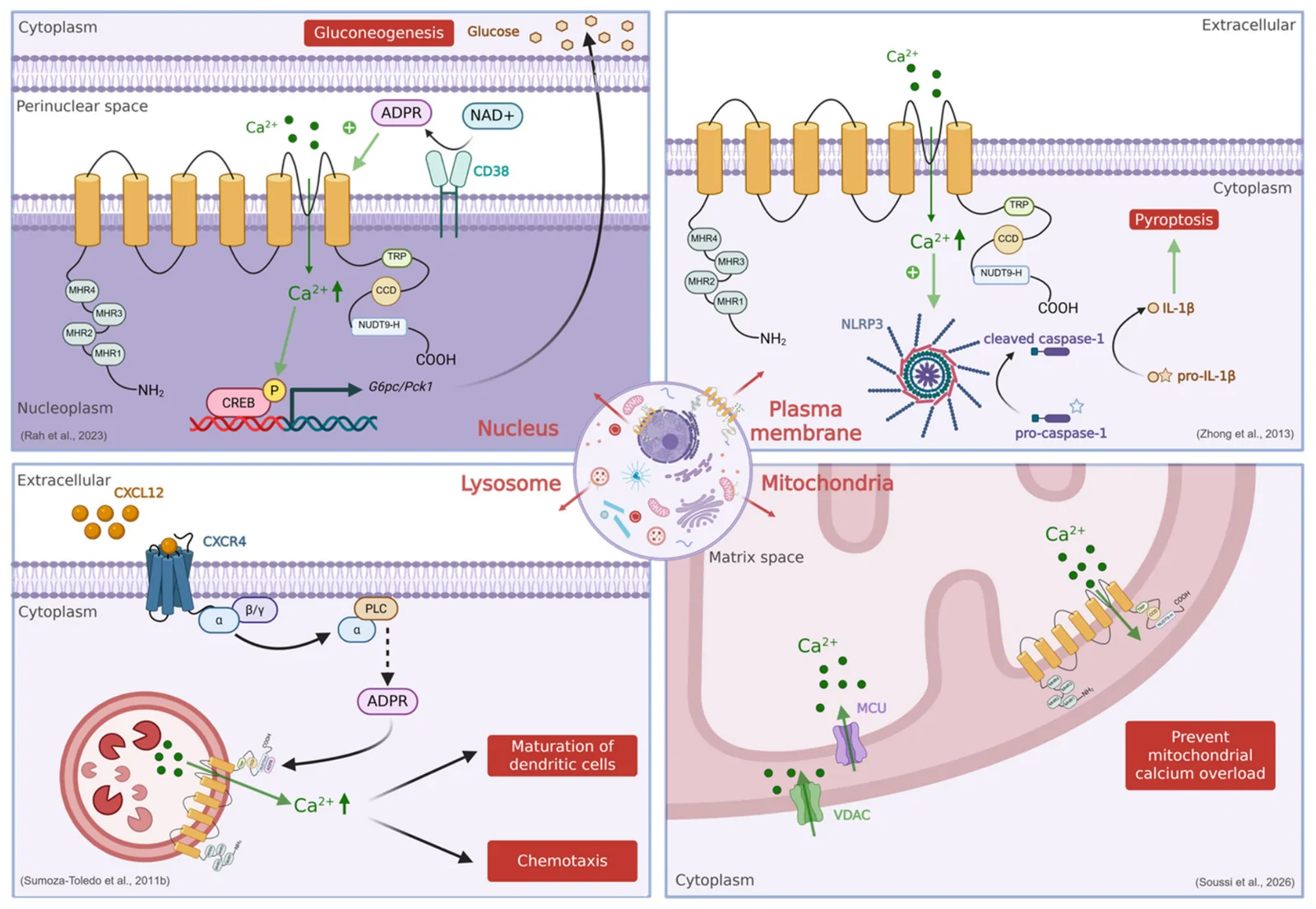
Subcellular compartmentalization of TRPM2 channel activity and associated signaling outcomes. (**Top left**) Nuclear envelope TRPM2 and gluconeogenic gene expression (adapted from [39]). CD38-mediated hydrolysis of NAD^+^ generates ADPR, which binds the NUDT9-H domain of TRPM2 at the perinuclear/nuclear envelope membrane, triggering Ca^2+^ influx into the nucleoplasm. The resulting rise in intranuclear Ca^2+^ promotes CREB phosphorylation, driving transcriptional activation of the gluconeogenic genes G6pc and Pck1 and subsequent glucose output. (**Top right**) Plasma membrane TRPM2 and NLRP3 inflammasome activation (adapted from [34]). TRPM2-mediated Ca^2+^ influx at the plasma membrane promotes NLRP3 inflammasome assembly, leading to caspase-1 autoproteolytic activation. Active caspase-1 cleaves pro-IL-1β into its mature form, culminating in pyroptotic cell death. (**Bottom left**) Lysosomal TRPM2 and dendritic cell function (adapted from [48]). CXCL12 binding to CXCR4 activates a Gα-dependent PLC signaling cascade, generating ADPR that activates lysosomal TRPM2. The resulting Ca^2+^ release from the lysosomal lumen is required for dendritic cell maturation and chemotactic migration. (**Bottom right**) Mitochondrial TRPM2 and Ca^2+^ homeostasis (adapted from [42]). Cytosolic Ca^2+^ enters the mitochondrial matrix through VDAC at the outer membrane and MCU at the inner membrane. TRPM2 activity at the inner mitochondrial membrane contributes to the regulation of matrix Ca^2+^ influx, limiting mitochondrial Ca^2+^ overload.

**Table 1:** Overview of TRPM2 subcellular localization, cellular effects, and signaling pathways. WB: Western Blot; IF: Immunofluorescence.

Localization	Cell Type	Effects	Signaling Pathway	Methods	Source(s)
Plasma membrane	Monocytes	Production of inflammatory cytokines, recruitment of neutrophils	Pyk2, Erk pathway, NF-κB	IF	[30]
	Macrophages	IL-1β secretion, pro-inflammatory response, and pyroptosis	NLRP3 inflammasome	Calcium imaging	[35,36]
	Hepatocytes, neurons and endothelial cells	Cell death via disruption of Ca^2+^ homeostasis	Ca^2+^ overload	IF (CD8)	[2]
	Hepatocytes	Dynamic trafficking of TRPM2	Oxidative stress-induced TRPM2’s trafficking	IF (cadherin)	[35]
Nucleus	Breast cancer	Increases cell proliferation	DNA damage response	WB (lamin B2)	[13]
	Oral squamous cell carcinoma	Promotes proliferation, migration, and Invasion	Oxidative stress, DNA damage response	IF (DAPI), cell fractionation (H3)	[37]
	Prostate cancer	Promotes proliferation and survival	Not clearly defined	IF (propidium iodide)	[38]
	Diabetes	Regulation of gluconeogenic gene expression	CD38/ADPR	IF (lamin B1), WB (lamin B1)	[39]
Mitochondria	Kidney injury	Promotes cell survival	AKT-mTOR pathway	IF (TOM20)	[40]
	Acute myeloid Leukemia	Promotes cell proliferation	Autophagy pathway	MitoSOX	[41]
	Breast cancer	Promotes cell survival	Preventing mitochondrial calcium overload	IF (Ds-Red2-Mito)	[42]
	Human corneal epithelial (HCE)	Promotes mitophagy	EGF/ROS-dependent TRPM2 activation	Mitophagy detection assay, JC-1 staining	[43]
	Pancreatic β cells and hippocampal neurons	Mitochondrial Zn^2+^ accumulation, mitochondrial fission	Lysosomal Zn^2+^– mitochondrial Zn^2+^–Drp1	IF (Mito-Cherry, MitoTracker), time-lapse confocal (FluoZin3), WB (Cyt-c)	[47,48,49,50]
Lysosome	Dendritic cells	Regulates dendritic cell maturation and chemotaxis	CXCL12/CXCR4-Ca^2+^ signaling	IF (LAMP-1)	[48]
	ß cells	Regulates ß-cell Ca^2+^ signaling	ADPR-dependent lysosomal Ca^2+^ mobilization	IF (LAMP-1)	[49]
	Aortic smooth muscle cells	Promotes autophagic degradation and lysosomal function	Lysosomal Ca^2+^-regulated autophagic flux	IF (LAMP-1)	[50]
	Mouse macrophages	Lysosomal Ca^2+^ release	ER–lysosome Ca^2+^ coupling; CaMKII/ASK1/ JNK–NLRP3 signaling	IF (LAMP-2)	[51]
Endoplasmic reticulum	Neuroblastome	Promotes cell proliferation and tumor growth	HIF-1α–dependent pathway	IF (Ds-Red-ER)	[52]

## Data Availability

No new data were created or analyzed in this study. Data sharing is not applicable to this article.
